# Pooling is an insufficient strategy to avoid healthcare staff to patient transmission of severe acute respiratory coronavirus virus 2 (SARS-CoV-2)

**DOI:** 10.1017/ice.2020.1340

**Published:** 2020-11-20

**Authors:** Jessica Lüsebrink, Verena Schildgen, Oliver Schildgen

**Affiliations:** Kliniken der Stadt Köln, Klinikum der Privaten Universität Witten/Herdecke, Institut für Pathologie, Köln (Cologne), Germany

*To the Editor—*Since the start of the coronavirus disease 2019 (COVID-19) pandemic, the need for diagnostic testing to detect infections and to interrupt infection chains has become more and more important. Especially in the healthcare sector, testing of employees is important to maintain basic medical care and to avoid transmissions of severe acute respiratory coronavirus virus 2 (SARS-CoV-2) from medical staff to patients, of whom many have an increased risk for serious clinical COVID-19 courses.

Our laboratory has been testing the staff of our hospital since the beginning of the pandemic. Like most laboratories, we have suffered from supply bottlenecks, especially with extraction kits. Therefore, we implemented pooling strategies that to respond to increasing test requests; such approaches have been discussed by other colleagues.^1^

Most of the specimens analyzed for our hospital staff screening have been throat washes from gargling with NaCl solution, as previously described.^2^ We reintroduced this well-known method^3^ due to the first shortage of swabs in the early phase of the pandemic in March 2020. A minority of orders received by our laboratory are for fast-track analyses using swabs and bronchoalveolar lavage (BAL) specimens.

In the first round, we started with pools of 10 samples (Table 1). Samples were prospectively tested both individually and mixed in a pool in parallel. A 300-µL sample was used for nucleic acid extraction with a Maxwell 16 Viral Total Nucleic Acid Purification Kit (Promega, Darmstadt, Germany), or 300 µL each of 10 different samples were mixed and 300 µL of the mixture was used for the extraction. The subsequent SARS-CoV-2 test was performed with the RealStar SARS-CoV-2 RT-PCR Kit (Altona Diagnostics, Hamburg, Germany). In total, 280 samples were tested, including throat washes (n = 247, 88.2%), swabs (n = 32, 11.4%), and bronchoalveolar lavages (n = 1, 0.4%). Overall, 8 samples (2.86%) were positive for SARS-CoV-2 by individual PCR assays. Of the 7 pools containing those samples, only 2 tested positive. For 2 pools, it was explainable that SARS-CoV-2 could not be detected because only a few copies were detected in the single PCR, but for the remaining 3 pools, we expected to be able to detect viral RNA, even with a dilution of 1:10 (ie, 1 pool contained 2 positive samples and tested negative). The test performance for the pooling strategy in comparison to the individual testing was as follows: sensitivity (29%), specificity (100%), positive predictive value (100%), and negative predictive value (19%).

One of the pools that tested positive for SARS-CoV-2 included a sample with an invalid PCR result (ie, the internal control was not amplified). This seemed to have a strong impact on the test result; the difference in the cycle threshold (Ct) value was much higher than expected in regard to the theoretic change in Ct values for the 1:10 dilution and in comparison to the second positive pool.

Because the pooling of 10 samples was unsatisfactory, we decreased the number of samples to 5 per pool. In total, 100 specimens (20 pools) were tested in this second round, but this time the pools were mixed together after a sample was tested positive and not in parallel, to minimalize the number of pools tested. The testing included pools that contained the samples that could not be detected in the larger pools and pools that contained invalid samples. Of 17 pools containing positive samples, 13 pools tested positive for SARS-CoV-2. Interestingly, the pools with negative results contained the same samples, which also could not be detected in the larger pools.

Invalid samples were pooled 3 times with negative samples. The PCR was inconspicuous and the amplification of the internal control was in range. Furthermore, 2 different invalid samples were each pooled with 1 positive and 3 negative samples and were compared to a pool containing another negative sample instead of the invalid one. This approach influenced the corresponding Ct values differently. Although we detected a difference of only 1 cycle for 1 sample, the other sample caused delays of 9 and 7 cycles in the respective genes. Obviously, this sample contained more or different PCR inhibitors, but the results show that samples containing inhibitors can have a crucial effect on pooled samples. The positive sample included a relative high amount of viral RNA. If the amount of RNA had been less, the test result for the pool presumably would have been negative.

However, considering that in the pools with 10 samples, only 2 of 7 pools (28.6%) tested positive as expected, and in the pools with 5 samples, 13 of 17 pools (76.5%) tested positive, pooling is not feasible in settings in which high sensitivity is crucial. Also, because samples with low viral RNA load and high Ct value could be infectious,^4,5^ a Ct of 30 or above should not be recommended, unlike previously published recommendations.^6^ Pooling strategies that decrease the sensitivity and increase the Ct also increase the risk of false-negative test results, which could lead to nosocomial transmission.

Finally, pools with 3 samples could be a proper solution; thus, we also analyzed whether this option would work in our current setting. Unfortunately, with a local prevalence of 6–8% positivity rate, the use of such pools with 3 specimens and the resolution of those pools would increase the use of filter tips for pipetting by one-third, which is currently not an option because of the worldwide interruption of delivery chains of filter tips.

**Table 1.** Overview of the Detailed Results of Pool Testing

Pool No.	Pool Result	Single Testing	Ct E Pool	Ct S Pool	Ct E Single Specimen	Ct S Single Specimen	ΔCt E	ΔCt S	Remark
**Pool with 10 specimens**									
1	–	–	–	–	–	–	–	–	
2	–	–	–	–	–	–	–	–	
3	–	–	–	–	–	–	–	–	
4	+	+	23.09	22.57	19.84	19.16	3.25	3.41	
5	–	–	–	–	–	–	–	–	
6	–	–	–	–	–	–	–	–	
7	–	–	–	–	–	–	–	–	
8	–	–	–	–	–	–	–	–	
9	+	+	35.04	35.73	28.16	27.7	6.88	8.03	Contained 1 invalid specimen
10	–	–	–	–	–	–	–	–	
11	–	–	–	–	–	–	–	–	
12	–	–	–	–	–	–	–	–	
13	–	–	–	–	–	–	–	–	
14	–	+	–	–	31.06	34.47	–	–	
15	–	–	–	–	–	–	–	–	
16	–	+	–	–	29.98	29.25	–	–	Contained 2 positive specimens
					27.15	26.63	–	–	
17	–	–	–	–	–	–	–	–	
18	–	–	–	–	–	–	–	–	Contained 1 invalid specimen
19	–	+	–	–	31.58	31.66	–	–	
20	–	–	–	–	–	–	–	–	
21	–	–	–	–	–	–	–	–	
22	–	–	–	–	–	–	–	–	
23	–	–	–	–	–	–	–	–	
24	–	–	–	–	–	–	–	–	
25	–	–	–	–	–	–	–	–	
26	–	+	–	–	38.48	–	–	–	
27	–	–	–	–	–	–	–	–	
28	–	+	–	–	35.29	–	–	–	
**Pool with 5 specimens**									
1	+	+	21.02	20.68	19.79	19.42	1.23	1.26	
2	+	+	25.92	38.3	31.53	32.21	−5.61	6.09	
3	+	+	22.18	21.49	22.58	21.15	−0.4	0.34	
4	–	–	–	–	–	–	–	–	Contained 1 invalid specimen
5	–	–	–	–	–	–	–	–	Contained 1 invalid specimen
6	–	–	–	–	–	–	–	–	Contained 1 invalid specimen
7	+	+	31.39	28.33	22.58	21.15	8.81	7.18	Contained 1 invalid specimen
8	+	+	23.43	22.83	22.58	21.15	0.85	1.68	Contained 1 invalid specimen
9	+	+	31.52	32.94	30.61	29.9	0.91	3.04	
10	+	+	17.24	16.58	16.67	16.62	0.57	−0.04	
11	+	+	16.24	16.61	19.6	17.8	−3.36	−1.19	
12	+	+	35.92	–	32.34	32.06	3.58	–	
13	+	+	30.89	31.47	29.19	29.89	1.7	1.58	
14	–	+	–	–	–	–	–	–	–
15	+	+	43.32	–	29.98	29.25	13.34	–	
16	–	+	–	–	27.15	26.63	–	–	
17	–	+	–	–	31.58	31.66	–	–	
18	–	+	–	–	38.18	–	–	–	
19	+	+	35.42	–	35.29	–	0.13	–	
20	+	+	35.67	42.88	29.98	29.25	5.69	13.63	Contained 2 positive specimens
					27.15	26.63	8.52	16.25	

Note. Ct, cycle threshold.
